# The Consequences of a Disruption in Cyto-Nuclear Coadaptation on the Molecular Response to a Nitrate Starvation in Arabidopsis

**DOI:** 10.3390/plants9050573

**Published:** 2020-05-01

**Authors:** Fabien Chardon, Gwendal Cueff, Etienne Delannoy, Fabien Aubé, Aurélia Lornac, Magali Bedu, Françoise Gilard, Stéphanie Pateyron, Hélène Rogniaux, Audrey Gargaros, Hakim Mireau, Loïc Rajjou, Marie-Laure Martin-Magniette, Françoise Budar

**Affiliations:** 1 Institut Jean-Pierre Bourgin, INRAE, AgroParisTech, Université Paris-Saclay, 78000 Versailles, France; gwendal.cueff@inrae.fr (G.C.); fabien.aube@ens-lyon.fr (F.A.); aurelia.lornac@unicaen.fr (A.L.); magali.bedu@bipm.org (M.B.); hakim.mireau@inrae.fr (H.M.); loic.rajjou@inrae.fr (L.R.); 2 Institute of Plant Sciences Paris-Saclay (IPS2), Université Paris-Saclay, CNRS, INRAE, Univ Evry, 91405 Orsay, France; etienne.delannoy@inrae.fr (E.D.); francoise.gilard@universite-paris-saclay.fr (F.G.); stephanie.pateyron@inrae.fr (S.P.); Marie-Laure.Magniette@inrae.fr (M.-L.M.-M.); 3 CNRS, INRAE, Institute of Plant Sciences Paris-Saclay (IPS2), Université de Paris, 91405 Orsay, France; 4 INRAE, UR BIA, F-44316 Nantes, France; helene.rogniaux@inrae.fr (H.R.); Audrey.Gargaros@evotec.com (A.G.); 5 INRAE, BIBS Facility, F-44316 Nantes, France; 6 UMR MIA-Paris, AgroParisTech, INRA, Université Paris-Saclay, 75005 Paris, France

**Keywords:** nutrient stress, natural variation, cytonuclear co adaptation, transcriptome, proteome, metabolome, *Arabidopsis thaliana*

## Abstract

Mitochondria and chloroplasts are important actors in the plant nutritional efficiency. So, it could be expected that a disruption of the coadaptation between nuclear and organellar genomes impact plant response to nutrient stresses. We addressed this issue using two *Arabidopsis* accessions, namely *Ct-1* and *Jea*, and their reciprocal cytolines possessing the nuclear genome from one parent and the organellar genomes of the other one. We measured gene expression, and quantified proteins and metabolites under N starvation and non-limiting conditions. We observed a typical response to N starvation at the phenotype and molecular levels. The phenotypical response to N starvation was similar in the cytolines compared to the parents. However, we observed an effect of the disruption of genomic coadaptation at the molecular levels, distinct from the previously described responses to organellar stresses. Strikingly, genes differentially expressed in cytolines compared to parents were mainly repressed in the cytolines. These genes encoded more mitochondrial and nuclear proteins than randomly expected, while N starvation responsive ones were enriched in genes for chloroplast and nuclear proteins. In cytolines, the non-coadapted cytonuclear genomic combination tends to modulate the response to N starvation observed in the parental lines on various biological processes.

## 1. Introduction

Assimilation of inorganic nitrogen (N) is crucial for plant growth and development and it requires energy and carbon (C) skeletons. When completely deprived of N supply, *Arabidopsis* (*Arabidopsis thaliana* L.) plants stop their above-ground growth, stimulate root growth and induce high affinity N uptake systems in roots [1,2]. In addition, they deeply modify their N and C metabolisms, and induce pathways that allow recycling and remobilization of N primary metabolites [3]. These metabolic adaptations are sustained by strong and organ-specific transcriptional responses [3,4,5,6]. Variations in the response to N supply were reported among *Arabidopsis* natural accessions, indicating that genetic variation modulates the physiological response of plants to N starvation [7,8]. Moreover, recent reports pointed to a possible link between metabolism plasticity, management of N pools and response to N availability in natural populations [9,10]. Mitochondria and chloroplasts are key players in plant N metabolism as in energy metabolism and C assimilation [11]. Indeed, the physiological response of plants to N starvation involves mitochondria- and chloroplast-located contributions, such as degradation of chlorophyll and chlorophyll apparatus, synthesis of flavonoid secondary metabolites, and production of metabolites necessary for nitrogen remobilization [2,12].

Mitochondria and chloroplasts are not only master players of cell metabolism. They also are “genetic organelles” that retained their own sets of genes from their bacterial ancestors [13], and their activity relies on the function of complexes of chimeric genetic origin (nuclear and organellar). Moreover, the persistence of highly conserved sets of genes in bioenergetic organelles was proposed to enable rapid adaptation of their electron transport chain complexes to environmental changes [14]. Indeed, environmental changes modify organelle gene expression, which in turn triggers retrograde signals to the nucleus, leading to nuclear transcriptional response [15]. In recent years, functional links between organellar retrograde signaling and plant response to environmental stresses have been substantially documented [16,17,18]. In parallel, many studies reported the role of organellar genetic variants in plant adaptation and crop performance [19,20]. Both nuclear and organelle genes jointly participate to the expression of organellar genomes and the constitution of multiproteic complexes [21,22], while genes in each compartment evolve at different paces [23,24]. Therefore, variants in organelle and nuclear genes have to be coadapted. Indeed, it has been shown that the phenotypic effects of organellar variants in plants and other eukaryotes mainly result from epistatic interactions with the nuclear genome [25]. Lines associating the nuclear genome from one parent with the organellar genomes from another parent—i.e., cytolines when both parents belong to the same species, alloplasmic lines when not—are valuable resources to study the phenotypical and molecular consequences of a disruption of cytonuclear coadaptation. Analyses of the transcriptomic response to a disruption of cytonuclear coadaptation are scarce, with only two reported studies in alloplasmic lines of wheat [26] and cytolines of maize [27].

In *Arabidopsis*, studies of cytolines or reciprocal recombinant inbred line populations showed that variations in cytoplasmic genomes, mainly in epistasis with the nuclear genome, influence many traits such as accumulation of defense metabolites [28], adaptive phenological traits [29], seed physiological features [30], and photosynthesis [31]. In the present work, we addressed the question whether transcriptomic, proteomic and metabolomic responses to N starvation rely on epistasis between the nuclear and organellar genomes. We used two natural accessions, namely *Ct-1* and *Jea*, and their reciprocal cytolines to explore the effects of a disruption of cyto-nuclear coadaptation on the molecular response to N nutrient stress. Among the core-collection used to develop series of cytolines in *Arabidopsis* [29], the *Jea*/*Ct-1* pair was found to have the highest genetic distance for the organelles and the second highest for the nucleus [32]. We observed that cytolines displayed a transcriptional signature of their novel genomic situation, which is different from the well described retrograde responses to organellar stresses. This signature modulates the responses of plants to N starvation, both at the metabolic and transcriptional levels.

## 2. Results and Discussion

### 2.1. Global Evaluation of the Modulation of Arabidopsis Response to N Starvation by a Disruption of Cyto Nuclear Coadaptation

#### 2.1.1. Experimental Design, Overall Phenotype, and Multi-Omics Strategy

**Figure 1:**
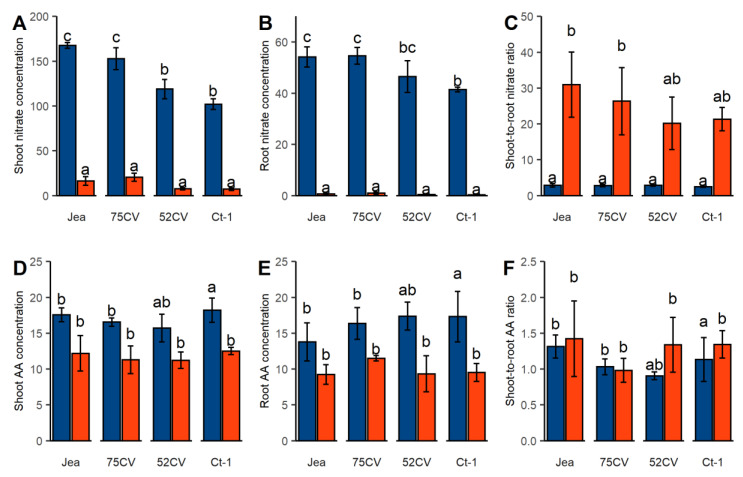
Nitrate and free amino acids concentrations (nmol.mg^−1^ FM) among the four *Arabidopsis* genotypes grown in the control and N starvation conditions. Bars show the average of nitrate concentration in shoot (**A**) and in roots (**B**), of the shoot-to-root nitrate ratio (**C**), of free amino acids in shoot (**D**) and in roots (**E**), and of the shoot-to-root amino acids ratio (**F**). Plants growing in control and N starvation are in blue and red, respectively. Error bars are SE (*n* = 6: 2 pools of four plants × 3 experiments). Different letters indicate values significantly different (Tukey’s test, *p* < 0.05).

We conducted three independent experiments where *Ct-1* and *Jea* natural accessions and their reciprocal cytolines, named 52CV (*Ct-1* nucleus and *Jea* cytoplasm) and 75CV (*Jea* nucleus and *Ct-1* cytoplasm) were submitted to two N supply conditions: one week N starvation and control N supply (see details in Material and Methods). In each experiment, each genotype was represented by eight plants per nutrition condition. We harvested five week-old plants at mid-day, roots and rosettes were separately weighted and immediately frozen. The average rosette fresh matter (FM) and root FM were similar between the cytolines and the parental lines in the two conditions (Figure S1). As expected, rosette FM was lower while root FM was higher in the N starvation condition compared to the control condition, resulting in a decrease of the shoot-to-root ratio. The reduction of the shoot-to-root ratio is a main feature of N starved plants [3,7]. We then measured nitrate (NO_3_^−^) and total free amino acids concentrations (Figure 1), which are the main pools of free N in cells [33], on ground rosette samples of pooled plants from each independent experiment. Small variations of nitrate concentrations were observed in shoot and roots between genotypes with nuclear *Jea* and *Ct-1* backgrounds under control N supply; the nitrate pools dropped in both shoot and root of all N starved samples (Figure 1A,B), leading to an increase of the shoot-to-root nitrate ratio (Figure 1C). The reduction of the free amino acid pools caused by N starvation was less important than the reduction of nitrate pools in shoots and roots (Figure 1D,E), and the shoot-to-root amino acid ratio remained stable (Figure 1F). The observed plasticity of nitrate and free amino acid pools was consistent with some previous reports [7,9]. This global survey confirmed that plants under N starvation condition responded in this experiment as previously described for *Arabidopsis* in similar nutrition stresses. At this level, we did not detect any obvious difference between the genotypes, in particular between the cytolines and the natural parental lines.

To investigate further the molecular responses to the nutrition stress in the shoot, and whether these responses were affected by new combinations of nuclear and organellar genomes, we performed a multi-omics approach. Using the rosette samples described above, we measured expression of 26,884 nuclear genes by mRNA hybridization on CATMA micro-arrays, and of 80 chloroplast (cp) and 31 mitochondrial (mt) genes by RT quantitative PCR, and quantified 665 proteins by LC/MS-MS and 81 metabolites by GC-MS TOF. Our experimental design allowed the evaluation of plant responses at three different molecular levels: gene expression (for the three genetic compartments), protein and metabolite contents. For all quantified entities, the same three-way ANOVA statistical model was applied (see 4.7 for details), considering nucleus and cytoplasmic genetic effects, nitrogen supply effect, and their pairwise and third order interactions. As interaction effects were taken into account, the effect of N starvation was tested averaged on all genotypes (contrast (i) in 4.7), and similarly the effect of the disruption of cyto-nuclear coadaptation (Cyt × Nuc interaction) was tested on the average of both N nutrition conditions (contrast (iv) in 4.7). Other effects—in particular the effect of the nuclear genetic background—were detected, but their analysis was beyond the scope of this report. The complete results of the statistical model and tests are available at [34]. 

In the present study, we chose to focus our analysis on the response to N starvation in cytolines compared to their natural parents. These results offered a first insight into whether the new cytonuclear combinations, despite the similar overall response, differentially coped the nutritional stress by balancing their molecular response. Because we had no previous knowledge about the molecular response to the new cytonuclear genomic combinations, we first checked whether the cytolines displayed a molecular signature similar to those described when the organelle functions are perturbed by chemical inhibition or by genetic mutations of organellar proteins (Section 2.1.2). Then, we explored the molecular responses to N starvation and addressed whether the cytolines, compared to their parents, displayed features that modified these responses (Section 2.1.3).

#### 2.1.2. Are Cytolines Under Organelle Stress?

We found 7153 nuclear differentially expressed genes (DEGs) under the influence of the Cyt × Nuc effect (averaged on both N nutrition), and strikingly most of them (7072) were repressed by the disruption of cyto-nuclear coadaptation, whereas only 81 genes were overexpressed in cytolines compared to parental lines (Table S1). This result indicates that nuclear genes whose expression is affected by new organelles-nucleus combinations were mainly down-regulated in the cytolines. In their transcriptomic analysis of maize cytolines, Miclaus et al. [27] observed 1179 DEGs affected in iso-nuclear cytolines compared to their nuclear parent but, in contrast to our study, they observed similar proportions of up- and down-regulated genes in the new genomic combinations compared to the parental line. Nine metabolites responded to Cyt × Nuc interaction, among which seven were more accumulated in cytolines compared to the parental lines (Table S3). A single differentially accumulated protein (DAP) responded to the Cyt × Nuc effect (Table S2). The very limited Cyt × Nuc effect on the proteome could be due to homeostasis processes for the maintenance of protein abundance through a fine-tune regulation of protein turnover and post-translational control.

Because cytolines possess genomic combinations of nucleus and organelles that did not coevolve, we wondered whether they expressed a transcriptional response related to that of plants submitted to organelle stresses usually used to study organellar retrograde regulation, such as treatments with inhibitors of organellar functions or mutations affecting these functions [35]. We thus compared the transcriptional response to cytoplasm exchange between *Ct-1* and *Jea* accessions to previously reported effects of mitochondrial or chloroplast stresses. On the basis of the lists established by Van Aken and Whelan [35], we found that only 2 out of the 14 genes identified as general markers for mt and cp dysfunctions (*AT1G66690* and *AT2G21640*), 3 out of the 12 genes identified as specific markers for mt dysfunction (*AT2G04030*, *AT2G40080* and *AT4G24175*), and 1 out of the 15 genes identified as specific markers for cp dysfunction (*AT5G27290*), were differentially expressed between cytolines and their parents (Table S4). In addition, all these genes were repressed in cytolines compared to their parents, whereas they are usually induced by organellar stresses. Therefore, the new cyto-nuclear genetic combinations of cytolines are unlikely to induce a response similar to the previously described organelle stress response. However, they do have a transcriptional signature, which may reflect a subtler response to their non-co-adapted genomic situation. In the previous maize study [27], a set of 96 DEGs were proposed to represent “key nuclear receptors of the retrograde signaling pathway”. Among the 82 *Arabidopsis* orthologs of this set, 67 were monitored in our experiment of which 19 were down-regulated in the cytolines compared to parental lines, while 46 were affected by N nutrition (Table S5). As suggested by these authors, the responsive DEGs to new cyto-nuclear genomic combinations very likely depends, at least in part, on the genetic polymorphisms between the nuclear and organellar donors of the cytolines under study.

#### 2.1.3. Overall Insight Into the Modulation of the Plant Transcriptional Response to N Starvation by the New Genomic Combinations in Cytolines

**Figure 2:**
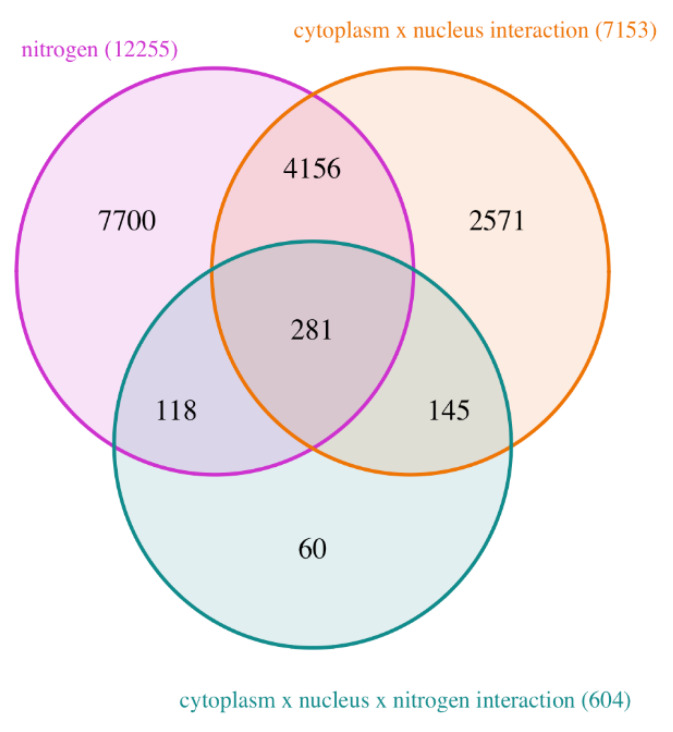
Venn diagram of DEGs affected by N nutrition or cytonuclear coadaptation. The total size of each set is indicated in brackets. “nitrogen”, DEGs responding to N nutrition (across all genotypes); “cytoplasm × nucleus interaction”, DEGs between cytolines and parent lines (across both N nutrition conditions); “cytoplasm × nucleus × nitrogen interaction”, DEGS responding to the three-order interaction.

In total, we obtained a clear and deep signature of N starvation, with 12,255 DEGs, 77 DAPs and 68 differentially accumulated metabolites (DAMs) affected by the N nutrition effect (averaged on all genotypes; Tables S1–S3). Because we were particularly interested in the effects that new cyto-nuclear combinations had on the plant response to N starvation, we focused our analysis on DEGs that were affected (i) by Cyt × Nuc × N interaction, which indicates a modification of the response to N starvation in the new cyto-nuclear genetic combinations, and (ii) by both N starvation (across all genotypes) and Cyt × Nuc interaction (across both nutrition conditions) effects. For these, the disruption of cyto-nuclear coadaptation either reinforces (when both effects act in the same direction), or attenuates (when the effects are in opposite directions) the response to N starvation. Indeed, 604 DEGs were affected by the Cyt × Nuc × N interaction, while 4437 genes (62% of the genes) that were differentially expressed in cytolines compared to natural parental accessions, were also responsive to N starvation across all genotypes (Figure 2). Among these 4437 DEGs, 281 were also affected by Cyt × Nuc × N interaction.

As a first insight into the cellular processes whose transcriptional regulation was affected by N starvation and the disruption of cyto-nuclear coadaptation, we evaluated whether DEGs were enriched in genes encoding proteins predicted to be located in specific cellular compartments (Table 1).

Whereas DEGs under N nutrition were significantly enriched in genes encoding proteins located in chloroplasts, those affected by Cyt × Nuc interaction were enriched in proteins located in mitochondria (Table 1). This difference between organellar locations of nuclear DEGs encoded proteins in response to N nutrition, was consistent with organellar DEGs, with 61 cp genes out of 80 (76%) differentially expressed under N nutrition, while it was the case for only 8 mt genes out of 32 (25%). In contrast, Cyt × Nuc interaction affected a single cp gene (namely *Photosystem II D2*) and no mt one.

This result confirms that (i) N nutrition stress had a strong effect on cp gene expression and on expression of nuclear genes encoding cp proteins, and (ii) that the disruption of cyto-nuclear coadaptation down regulated the nuclear genes encoding mt proteins, but did not modify the expression of mt genes. It is noteworthy that the maize study have shown that the set of DEGs responding to the disruption of cyto-nuclear coadaptation in maize cytolines encoded more cp-located proteins than mt-located ones [27].

Both the DEGs under N nutrition and DEGs responding to Cyt × Nuc interaction were enriched in genes encoding nuclear located proteins (Table 1). Moreover, among DEGs under N nutrition those also affected by the disruption of cyto-nuclear coadaptation were further enriched in nuclear protein coding genes. This suggests a modulation of the response to N starvation in the cytolines compared to parents through a tuning in gene regulation. DEGs affected both by N nutrition and Cyt × Nuc interaction were also further enriched in genes encoding proteins located at the plasma membrane, compared to DEGs under N nutrition.

**Figure 3:**
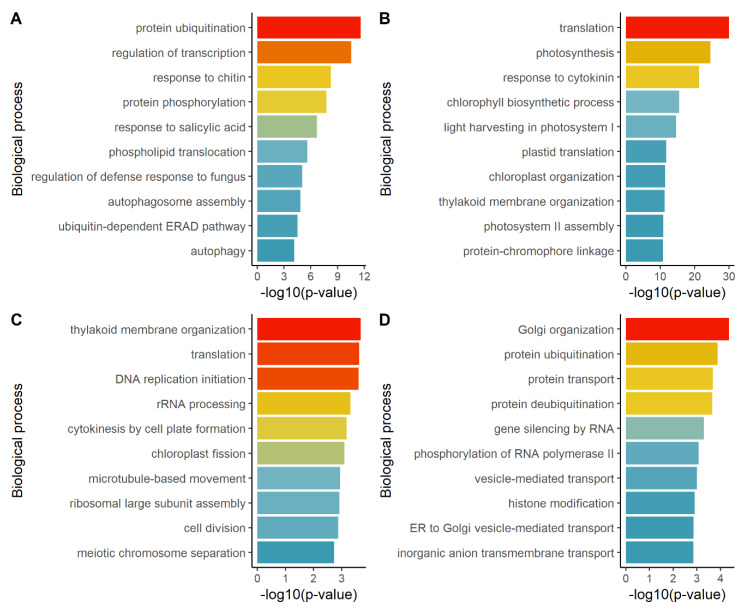
Enriched Gene Ontology (GO) terms in biological processes for DEGs responsive to N starvation and Cyt × Nuc interaction. The top 10 significantly enriched GO categories (*p* < 0.05) for each analysis, classified into different functional categories according to the GO term enrichment analysis for “biological processes”. Panels **A** and **B** show enrichments in the DEGs up regulated and down regulated in N starved plants, using all genes examined as reference. Panels **C** and **D** show enrichments in biological processes in DEGs responsive to both N starvation and Cyt × Nuc interaction with identical (**C**) or opposite (**D**) direction effects, using all DEGs responsive to N starvation as reference.

As N starvation response has been previously studied, we verified that N-starved plants displayed the expected signature for this stress by performing separate Gene Ontology (GO) enrichment analysis for “biological processes” on up-regulated and down-regulated genes under N starvation in our experiment. DEGs up-regulated under N starvation clustered in several processes related to the regulation of transcription, post-translational modification of proteins (i.e., protein ubiquitination and protein phosphorylation), response to chitin and salicylic acid, autophagy process and phospholipid translocation (Figure 3A). DEGs down-regulated under N starvation clustered in processes related to translation, response to cytokinin and plastid functions, such as photosynthesis and plastid organization (Figure 3B). The enrichments of GO terms are coherent with the previous studies characterizing the transcriptional response to N starvation [3,4,5,6].

Then, we identified biological processes involved in the N starvation response which were modified in cytolines compared to parent lines, by performing the GO enrichment analysis on the 4437 DEGs responsive to both N starvation and to Cyt × Nuc, either with identical or opposite directions, taking as reference the set of N nutrition responsive DEGs. The most enriched terms for DEGs with identical direction effects under N starvation and Cyt × Nuc interaction were similar to the GO terms enriched for down-regulated genes under N starvation (Figure 3C), which indicates that the genetic situation in cytolines induces a transcriptional response similar to the response to N starvation for these biological processes. These genes were mostly under expressed in N starved plants compared to controls and in cytolines compared to parents. The enriched terms for DEGs with opposite direction effects under N starvation and Cyt × Nuc interaction were related to the Golgi organization, protein ubiquitination, protein transport, vesicle-mediated transport and gene silencing (Figure 3D). These genes were mostly down-regulated in cytolines compared to parents and upregulated in N starved plants compared to controls, which suggests that genes of these biological processes tend to be repressed in the new genetic combinations of cytolines whereas they are induced by N starvation.

The same type of GO enrichment analysis on the 604 DEGs responding to the third order Cyt × Nuc × N interaction did not return any hit.

This overview suggested that several important responses to N starvation were tuned differently in cytolines and in parents. We therefore examined some of the cellular processes and pathways involved in the response to N starvation to evaluate how the disruption in the genomic coadaptation modulated them.

### 2.2. The Modulation of the Response to N Starvation in Cytolines for Specific Cellular Processes/Pathways

The overall analysis conducted in the previous section indicated that the disruption of cyto-nuclear coadaptation modified the response to N starvation in specific pathways. In this second section, we investigated the main components of the response to N starvation, identified in the GO analysis as sensitive to the Cyt × Nuc interaction. To give an idea of the importance of the disruption of the cyto-nuclear coadaptation, we first gave the global effect of the N starvation and then the effect of the Cyt × Nuc interaction on different biological processes.

#### 2.2.1. Cyto-Nuclear Coadaptation Fine-Tunes the Response of Central Metabolism to N Starvation

**Figure 4:**
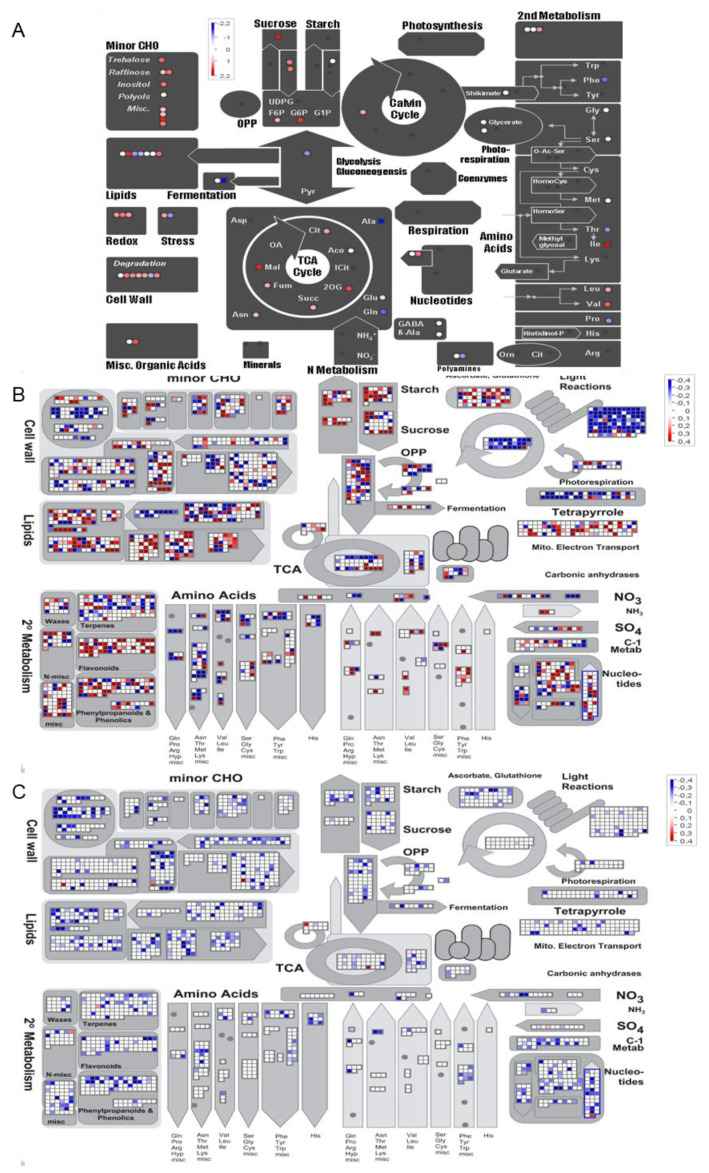
MapMan representations of the effects of N starvation on (**A**) metabolite amounts and mRNA and (**B**) mRNA accumulation for genes involved in primary metabolism, and (**C**) of the effects of Cyt × Nuc interaction on mRNA accumulation for genes involved in primary metabolism. Red and blue symbols mean enrichment or depletion of molecules/mRNA under N starvation or in cytolines compared to parents, based on the calculated contrasts following the formulas (i) and (iv) (see Section 4.7).

MapMan software v6.3 [36] was used to gain insight into the biological processes affected by N starvation and Cyt × Nuc interaction. Figure 4 illustrates the DAMs and DEGs involved in the central metabolism (see complete lists in Tables S1 and S3). Under N starvation, most of the individual amino acids decreased whereas the sugars, such as glucose, fructose, raffinose and galactose, were accumulated (Figure 4A). We also noticed that pyruvate amount was reduced while most of organic acids were increased under N starvation. The changes in individual amino acid amounts were coherent with our measurement of total free amino acids in shoot (Figure 1), and are typical of N starvation [3]. Changes in mRNA amounts of genes coding for enzymes involved in the central metabolism (Figure 4B) were in accordance with the metabolite amount variations in sugars, amino acids and organic acids pathways (Figure 4A). The Cyt × Nuc interaction affected also the contents of sugars, increasing mannose and galactose but reducing melibiose and raffinose, and some organic acids content, increasing alpha-ketoglutaric, 2-hydroxyglutaric and citramalic acids (Table S3). Furthermore, raffinose is under Cyt × Nuc × N interaction, revealing that the increase of this sugar in response to N starvation is higher in the parental lines than in the cytolines (Table S3). Raffinose accumulation under abiotic stresses, in particular under N starvation, has been previously reported [3,37]. Raffinose is synthetized in the cytosol and transported into plastids to protect thylakoid membranes, contributing to PSII integrity and acting as a potential ROS scavenger [38,39].

The genes for the nitrate assimilation pathway, including nitrate transporters, were mainly repressed by N starvation (Figure 4B), most likely due to the absence of nitrate and to the rise of sugar content that represses the transcription of these genes [40]. Three genes encoding nitrate transporters were induced by N starvation: *NRT2.5* (*AT1G12940*) and *NAR2* (*NRT3.1*; *AT5G50200*) that encode high affinity nitrate transporters previously described as main actors in N starvation response [40], and *NRT1:4* (*AtNPF6.2*; *AT2G26690*) that encodes a petiole-specific nitrate transporter [41]. The Cyt × Nuc interaction reduced the mRNA accumulation of two nitrate transporters: NRT1.2 (*AtNPF4.6; AT1G69850*) a both nitrate and ABA transporter [42] and NRT2.7 (*AT5G14570*) a vacuolar high affinity nitrate transporter [43]. The induction of genes encoding enzymes well known to be involved in ammonium assimilation, such as *GLN1.4* (*AT5G16570*), *GLU1* (*AT5G04140*) and *GDH1* (*AT5G18170*) [44,45], suggests that processes for and amino acids recycling were engaged in starved plants at the harvest, likely fueled by protein catabolism, which explains the AA content at 50% of the optimum observed in the control condition (Figure 1D). Genes coding for several enzymes of this pathway were reduced in the cytolines compared to the parental lines, such as *GLN1.4* (*AT5G16570*), *GLN1.5* (*AT1G48470*), *GLU2* (*AT2G41220*) and a putative glutamate dehydrogenase (*AT1G51720*) (Figure 4C).

The mRNA amounts of most genes involved in the photosynthesis apparatus, photorespiration, Calvin cycle, and tetrapyrrole synthesis pathway decreased under N starvation (Figure 4B), in accordance with the accumulation of known repressors of these pathways, namely glucose 6-phosphate and trehalose 6-phosphate [46]. In addition, the genes encoding enzymes involved in starch metabolism were activated by N starvation (Figure 4B). Starch turn-over has been described as a regulator of the plant growth [47]. The regulation of starch turn-over might explain the limitation of plant shoot growth observed in N starvation (Figure S1). However, several of these genes involved in starch turnover were repressed in cytolines compared to parent lines (Figure 4C), suggesting that this response was limited in cytolines. Genes involved in flavonoid biosynthesis were enhanced, suggesting the accumulation of pigments in N starved plants, as previously reported [48]. This was also in accordance with the observation of a few more yellow leaves in N starved plants than in control plants (Figure S1). In addition to the reduction of the pyruvate amount, we noticed a decreased of the transcriptional activity of genes involved in the geranylgeranyl diphosphate pathway, two precursors of carotenoids localized the cytosol and the mitochondria [49], and the reduction of mRNAs coding for geranylgeranyl and carotenoid biosynthetic enzymes, under N starvation. The analysis of DAPs revealed that the variations of transcript levels under N starvation were associated with changes in corresponding protein abundance for starch turnover and tetrapyrrole biosynthesis (Table S2).

#### 2.2.2. Cyto-Nuclear Coadaptation Acts on Major Actors of N Recycling Process in Response to N Starvation

N starved plants exhaust their vacuolar nitrate and amino acid stocks within few hours. Then their unique source of N compounds comes from the recycling and remobilization of macromolecules [3]. The ubiquitin/26S proteasome pathway and the macro-autophagy (called autophagy hereafter) system are the two main molecular mechanisms involved in the recycling of macromolecules. In the ubiquitin/26S proteasome pathway, proteins are marked with a poly-ubiquitin chain to serve as effective substrates for cleavage by the 26S proteasome whereas the autophagy system engulfs and sequesters unwanted cytoplasmic constituents into cytosolic double membrane vesicles to drive them to the central vacuole [50].

**Figure 5:**
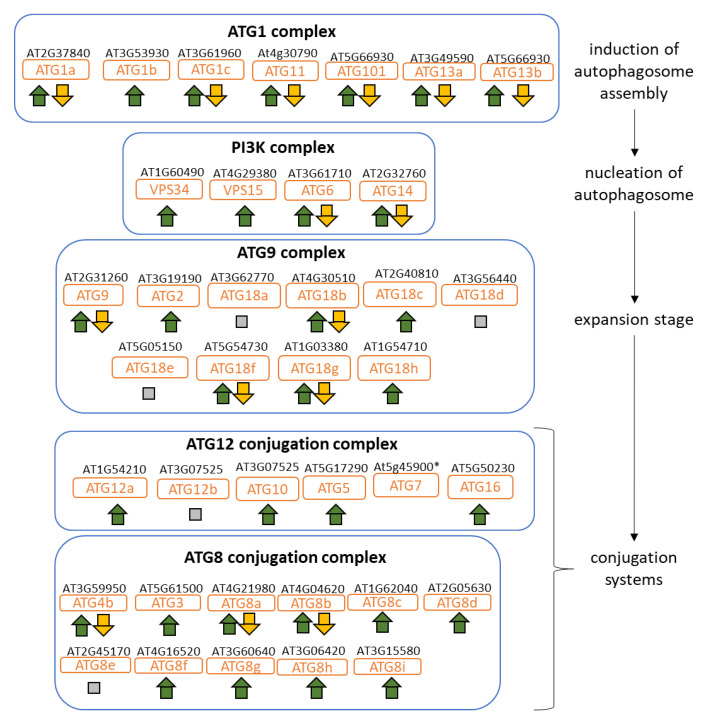
Effects of N starvation and disruption of cyto-nuclear coadaptation on autophagy-related genes. The steps of autophagosome assembly are indicated on the right; vertical arrows indicate the time progression. The complexes involved in each step and their composition are indicated on the left, in frames. The symbols indicate the results of the relevant ANOVA tests: grey, not significant; green, responsive to N starvation; yellow, responsive to disruption of cytonuclear coadaptation; the direction of the arrow indicates the direction of the effect; * non analyzed gene due to CATMA probe target polymorphic between the parental lines (details in Material and Methods section). None of the proteins encoded by these genes was quantified in the experiment.

In N limiting conditions, autophagy is highly efficient in the recycling of proteins and organelles, and has an essential role in maintaining free amino acid pools [51,52]. In this experiment, autophagy was one of the 10 most enriched biological processes in upregulated DEGs in N starved plants (Figure 3A). We noticed that trehalose, one of the inducers of autophagy [53], over-accumulated in starved plants. In *Arabidopsis*, 38 autophagy-related (*ATG*) genes are involved in the autophagy system [54]. We examined more specifically the *ATG* genes for their responses to the effects of interest (Figure 5). As expected, almost all *ATG* genes involved in autophagosome machinery are up regulated in starved plants. However, the genes involved in early steps, particularly in the induction of autophagy, were less expressed in cytolines compared to the parental lines, suggesting that the autophagy might be less intense in the plants with new cyto-nuclear combinations than in parental lines in the N starvation condition.

The ubiquitin/26S proteasome pathway involves more than 1300 genes, ~5% of the proteome [55]. In this pathway, the ubiquitin serves as a reusable tag for selective protein breakdown. It is covalently attached to target proteins by a cascade enzymatic system consisting of Ub-activating (E1), conjugating (E2), and ligating (E3) enzymes. Once a conjugate is assembled, bearing a chain of multiple ubiquitin, it is recognized by the 26S proteasome and degraded. In plants, four E3 types have been described, based on subunit composition and mechanism of action [Homology to E6AP C Terminus (HECT), Real Interesting New Gene (RING)/U-Box, a complex of Skp1, CDC53, and F-box protein (SCF), and anaphase-promoting complex (APC)] [56,57]. In addition, proteins having a BTB (Bric-a-brac/Tramtrack/Broad complex) domain have been identified as proteins interacting with the SCF complex [58]. We investigated the response of 640 genes encoding for proteins of the E1, E2 and E3 complexes; 107 of them were not analyzed due to polymorphisms between the two parental lines on the CATMA microarray probe sequence. Among the 533 genes measured, 302 responded to N starvation, 271 upregulated and 31 downregulated (Table S6). Genes encoding E1 and E2 proteasome complex subunits were all affected by N starvation or Cyt × Nuc interactions. Among these 17 genes, six responded to both effects and were induced by N starvation but less expressed in cytolines compared to the parental lines. We recorded 23 DEGs in Cyt × Nuc × N interaction: one encodes a BTB protein (*BT1*, *AT5G63160*), and 22 encode E3 RING/U-Box type proteins. BT1 is a short-lived nuclear-cytoplasmic protein that is targeted for degradation by the 26S proteasome pathway [59].

Interestingly, BT1 was described as major regulator of Nitrogen Use Efficiency (NUE) in *Arabidopsis* and rice, regulating nitrate uptake and playing a role in transcriptional regulation in response to nutrient status [60,61]. In this experiment, cytolines repressed its expression in response to N starvation while the natural parents induced it. The roles of the 22 RING proteins are less known, except for three noteworthy of them. RHA2B (*AT2G01150*) regulates ABA signaling and drought tolerance [62,63]; RGL4 (*AT1G79380*) encodes an essential upstream modulator of JA signaling in response to various stimuli [64] and of iron sensing [65]; NLA (*AT1G02860*) is a major actor in the response to low-nitrogen conditions in *Arabidopsis* which interacts with ORE1 in the nucleus to induce leaf senescence during N deficiency [66] and regulates the phosphate homeostasis in interactions with microRNA *miR827* [67]. A single protein of E1 complex, UBIQUITIN-ACTIVATING ENZYME 1 (ATUBA1), was quantified in this experiment. The ATUBA1 amount did not vary although the transcriptional activity of its gene (*AT2G30110*) was lower in cytolines compared to parental lines, suggesting a compensation effect at the level of translation or protein turnover. We concluded that the N starvation induces the ubiquitin/26S proteasome pathway but the signal made by the two major actors of N response, BT1 and NLA, is reduced in cytolines compared to the parental lines.

**Figure 6:**
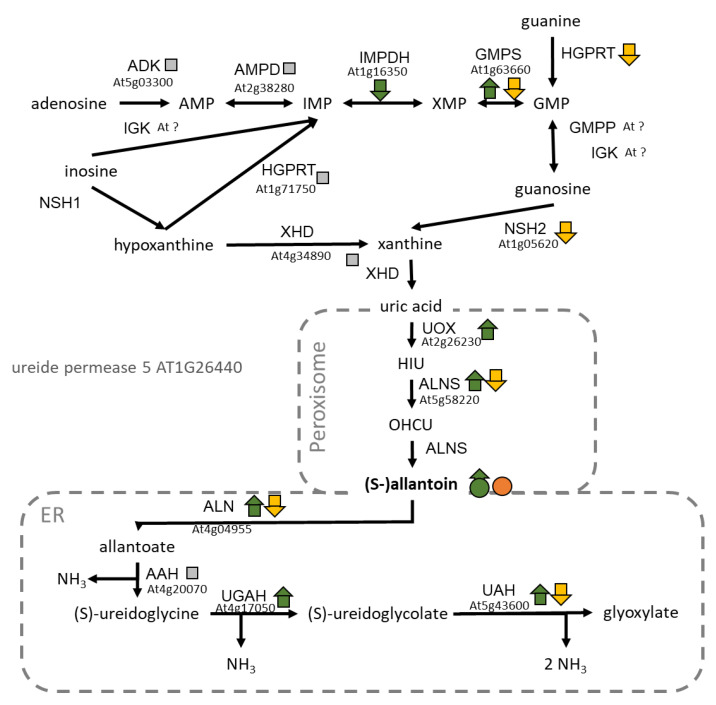
Effects of N starvation and disruption of cyto-nuclear coadaptation on allantoin synthesis and degradation. Simplified pathway of allantoin biosynthesis and degradation [68]. The symbols indicate the results of the ANOVA tests. Squares are for DEGs, circles for differentially accumulated metabolites (DAMs); grey, not significant; green, responsive to N starvation; yellow, responsive to disruption of cytonuclear coadaptation; orange, significant Cyt × Nuc × N interaction; the direction of the arrow indicates the direction of the effect. None of the other metabolites was quantified. None of the quantified proteins was affected by the relevant effects.

Allantoin is an intermediate metabolite of purine catabolism which may contribute to N recycling to produce cellular ammonium [68,69]. Allantoin was one of the ten metabolites affected by the Cyt × Nuc × N interaction in our experiment. All genotypes accumulated more allantoin under N starvation than in control conditions, but this effect was enhanced in cytolines compared to parental accessions. Examination of the transcriptional response of the degradation pathway of purines (Figure 6) suggested that (i) the N starvation induced it, but (ii) in cytolines, expression of both synthesis and degradation enzymes was lower compared to parents. In rice, the allantoin level in shoots is known to increase during N starvation and to decrease when N sources are re-applied to the plants [70]. We hypothesized that the higher accumulation of allantoin in our cytolines reflects either a less efficient purine recycling or a higher N response for purine degradation in the cytolines compared to their parental lines.

#### 2.2.3. Disruption of Cyto-Nuclear Coadaptation Perturbs the Adaption of Lipid Biosynthesis and of Energy Metabolism to N Starvation

The single protein affected by the Cyt × Nuc × N interaction, ENOLASE 2 (ENO2), was more accumulated under N starvation in cytolines than in the parental lines (Table S2). ENO2 was described as a bifunctional enolase localized to the cytoplasm and to the nucleus [71,72]. ENO2 is the cytosolic isoform of the enzyme converting 2-phospho-D-glycerate into phosphoenolpyruvate (PEP). PEP is an important energetic metabolite that contributes to glycolytic ATP production through the transfer of its phosphate group to ADP by the pyruvate kinase. PEP is also a precursor of oxaloacetate and acetyl-coA, entry points in the TCA cycle and the fatty acid biosynthesis pathway. As an important substrate for the phosphoenolpyruvate carboxylase (PEPC), PEP is essential for the anapleurotic role of PEPC in the biosynthesis of amino acids. Because PEP is such an important metabolite, the modulation of ENO2 by the Cyt × Nuc × N interaction suggests that the energy metabolism is differently affected by N starvation in cytolines compared to natural accessions. The function of ENO2 in the nucleus also relates with the plant response to stress. Indeed, the ENO2 protein sequence contains both DNA-binding and repression domains [73]. ENO2 was shown as a repressor of *STZ/ZAT10* expression, which encodes a transcription factor with two Cys(2)/His(2)-type zinc-finger motifs that act as transcriptional repressors for oxidative stress responses [71].

**Figure 7:**
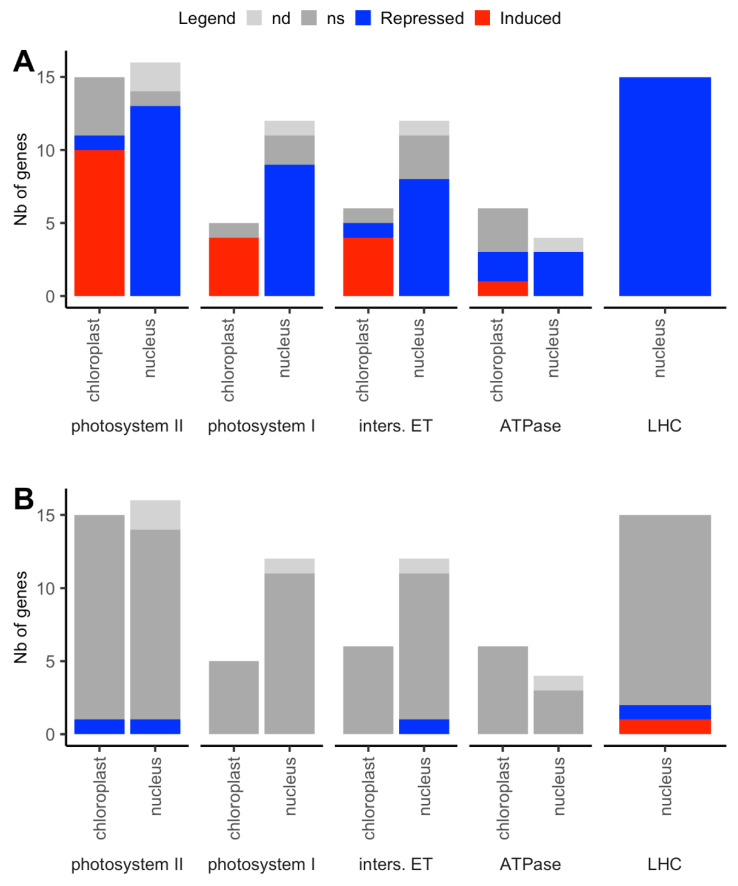
Transcriptional responses to N starvation (**A**) and to the disruption of cytonuclear coadaptation (**B**) of genes encoding the proteins involved in the photosynthetic apparatus. The genes are classified by components of the different complexes: photosystem I, photosystem II, intersystemic electron transport (inters. ET), ATP synthase (ATPase), light harveting complexes (LHC) (See also Table S7. Colors show genes significantly up- and down-regulated by N starvation in red and blue, respectively; non-significant (ns) in dark grey and non-detected (nd) in light grey.

The lack of nitrate suppresses the use of C skeletons from photosynthesis for its assimilation [11]. In addition, since up to 75% of the inorganic N present in mesophyll cells is located in chloroplasts, mainly as RubisCO [33], enzymes of photosynthesis apparatus have been reported as a major source for N recycling in leaf cells, in particular during leaf senescence [73]. Indeed, photosynthesis and assembly of the photosynthetic apparatus were represented in three of the top ten biological processes enriched in nuclear DEGs repressed by N starvation (Figure 3). Both cp and nuclear-encoded proteins are required to form the photosynthetic complexes, but to function optimally, plastid metabolism must be tightly integrated with that of the whole cell [74]. We examined the effects of N starvation (Figure 7A) and disruption of cyto-nuclear coadaptation (Figure 7B) on the expression of both cp and nuclear genes for photosynthetic complexes (Table S7). Among 85 measured genes (31 in the chloroplast and 54 in the nucleus), 71 were affected by the nitrogen supply. Surprisingly, while all the 48 nuclear genes concerned were repressed in starvation, most of the cp genes (19) were upregulated: the transcriptional response of plastid genes seemed to be disconnected from that of nuclear genes under N starvation. Similar contrasted responses to N starvation have been reported for cp and nuclear genes for PSII and PSI in Chlamydomonas (*Chlamydomonas* *reinhardtii*), where the reaction center core subunits (encoded by plastid genes *psbA* and *psbB* for PSII; *psaA* and *psaB* for PSI) are more stable than the peripheral proteins and LHCII antenna proteins under N starvation [75]. In our experiment, none of the proteins encoded by these DEGs and quantified in the study was variable, indicating a compensatory effect at the translational of post-translational levels of observed variations in gene expression. Very few genes of the photosynthetic complexes were affected by the Cyt × Nuc (5 DEGs) or Cyt × Nuc × N interactions (two DEGs), such as *psbD* (*ATCG00270*), affected by all the considered effects, for which the induction by N starvation was more important in parental lines than in the cytolines.

The redox state of the photosynthetic electron transport chain and the amount of sugars have been shown to regulate the expression of plastid-encoded photosynthetic genes [76,77,78]. In our experiment, plants accumulated high levels of sugars but genes coding for enzymes (GSH1 and GR1) involved in the metabolism of glutathione, an important cellular redox buffer, were not significantly enhanced by N starvation.

Because *de novo* fatty acid biosynthesis mainly takes place in the plastidial compartment from acetyl CoA, which is a direct product of photosynthesis [79], we investigated the effect of N starvation and the disruption of cyto-nuclear coadaptation on lipids production and management. N deprivation is well known to affect chlorophyll content, photosynthesis rate and the abundance of thylakoid membranes in chloroplasts [80]. Both photosynthesis, plastid organization and thylakoid membrane organization were in the top ten biological processes enriched in DEGs repressed by N starvation (Figure 3). In addition, the composition of the galactolipids of photosynthetic tissues were reported to vary extensively in N deprived plants and the amount of monogalactosyldiacylglycerol (MGDG) content decreased whereas the digalactosyldiacylglycerol (DGDG) increased [80]. In plants subjected to N starvation, we observed a decrease of several basic fatty acids (Table S3), such as palmitic and stearic acids. We did not measure the different lipids in our samples, but we investigated 778 genes known to be involved in fatty acid and lipid metabolism [81]. Massive decrease of mRNA abundance was observed for genes involved in fatty acids synthesis, strongly suggesting that lipid production was affected by N starvation in our condition (Table S8). In contrast, we also observed that several genes coding for (i) lipid desaturases, like stearoyl-ACP desaturase, (ii) lipid trafficking, like amino-phospholipid ATPase (*AT5G04930*) and phospholipid translocase (*AT1G59820*), (iii) sphingolipid biosynthesis, as palmitoyltransferase and ceramide synthase, (iv) lipases and (v) lipid signalization were induced, suggesting that the lipid management was affected by nitrate starvation. We found 176 of the 778 listed genes were affected by the Cyt × Nuc interaction, with lower expression in the cytolines than in the parental lines except for a monoacylglycerol lipase (MAGL, *AT1G73480*) which was induced in the new genetic combinations. The DEGs responding to the cytoplasm exchange were mainly involved in phospholipid signaling, oxylipin metabolism and phospholipases D. Only 20 genes were impacted by the Cyt × Nuc × N interaction and for 17 of them the response to N starvation was lower in the cytolines than in the parental lines for those induced by starvation, and higher for those repressed by starvation. Altogether, the results suggest that the fine tuning of the lipid biosynthesis that occurs during the N starvation was perturbed by the disruption of cyto-nuclear coadaptation.

**Figure 8:**
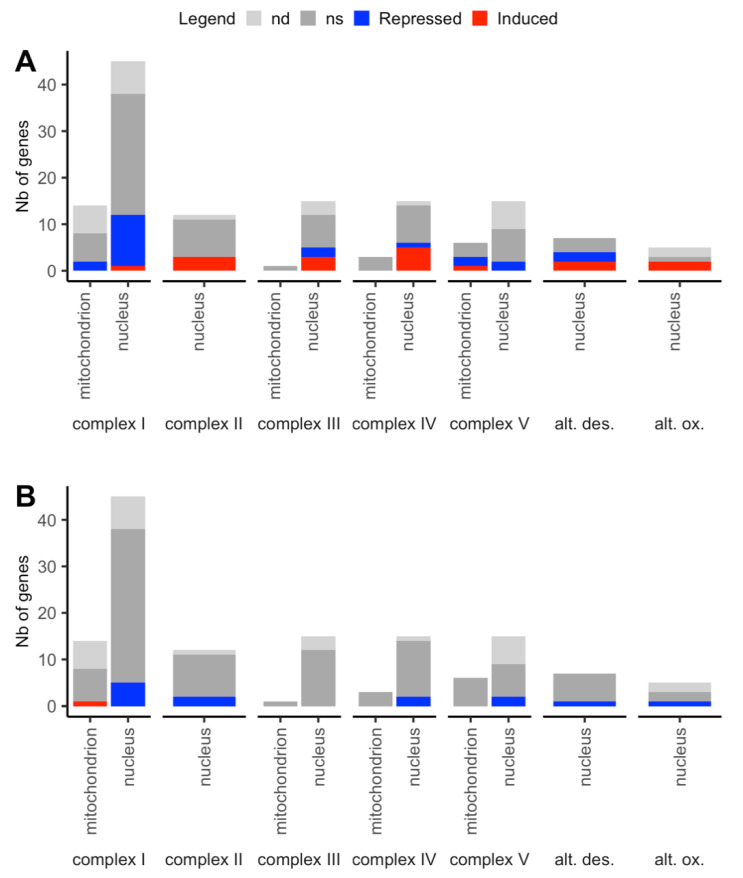
Transcriptional responses to N starvation (**A**) and to the disruption of cyto-nuclear coadaptation (**B**) of genes encoding the proteins involved in the mitochondrial respiration. The genes are classified by components of the mitochondrial electron transport chain complexes (complex I, II, III, IV and V), alternative NADH dehydrogenases (alt. des.) and alternative oxidases (alt. ox.) (See also Table S9). Colors show significant genes up- and down-regulated by N starvation in red and blue, respectively; non-significant (ns) in dark grey and non-detected (nd) in light grey.

Mitochondrial respiration is the fundamental energy-converting process, generating the ATP needed for cell maintenance and growth, and placing mitochondria at the center of stress signaling networks and metabolic homeostasis pathways [82]. Proteins encoded by the nuclear genome (94 genes) and the mitochondrial genome (15 genes) form the mitochondrial electron transport chain. Assembly of a functional and responsive respiratory chain requires the coordinated expression of these two sets of subunit-encoding genes located in distinct genetic compartments [83]. We examined the transcriptional response of 138 genes (114 nuclear and 24 mt genes) involved in mitochondrial respiration. Overall, the expression of genes encoding subunits of the mitochondrial respiration complexes and alternative pathways was much less affected by N starvation than those of genes encoding photosynthetic complexes (Figure 8A) and not more by the new cyto-nuclear genetic situation of cytolines (Figure 8B).

Only 38 genes were affected by N starvation, 22 down-regulated and 16 up-regulated, among which were four alternative NADH dehydrogenases and isoforms of the alternative oxidase A. Varying complex I and complex V subunit encoding genes were mainly down-regulated by N starvation, whereas those in complexes II, III and IV were mainly up-regulated. Similar results were obtained on the abundance of proteins of the mitochondrial electron transport chain. Among the 10 proteins measured in the experiment, the abundance of seven proteins was not modified between the two nutritive conditions. The two quantified complex II subunits SDH1 (*AT5G66760*, upregulated DEG), SDH5 (*AT1G47420*, transcription not analyzed in the experiment) were more accumulated in starved plants than in controls. We concluded that unlike the reduction of mRNAs coding for the photosynthetic apparatus, the relative mRNA abundances for respiratory complexes were maintained under the N starvation. Our results are consistent with similar observations reported in Chlamydomonas [75]. The new genetic situation of cytolines did not substantially affect subunits of the respiratory complexes. It is noteworthy that among the 14 DEGs responding to the Cyt × Nuc effect, all 13 nuclear DEGs were down expressed in cytolines, whereas the mt *nad4* gene (*NADH dehydrogenase subunit 4*, *ATMG00580*) was more expressed in cytolines than in parents. Furthermore, the alternative oxidases (AOXs; which bypass complexes III and IV of the electron transport chain) and two type II NAD(P)H dehydrogenases (which bypass complex I) were overexpressed under N starvation (Table S9); the alternative NADH dehydrogenase NDB2 (*AT4G05020*, upregulated DEG) was also more accumulated in starved plants than in controls. The alternative pathway is part of the mitochondrial reactive oxygen species-scavenging network [84] and was proposed to balance carbon metabolism and electron transport [85]. The expression of these alternative NADH dehydrogenases and alternative oxidases was reported to be repressed by nitrate [85] and induced by mitochondrial stresses [84,86]. Here, one of the two DEGs encoding the isoforms of the AOX1a induced by N starvation was repressed in cytolines compared to parental lines.

TCA cycle metabolites are important for the intercompartmental exchange in plant cells and were proposed to have a signaling function for the status of mitochondrial bioenergetic processes [87]. We observed that malate and glycine were affected by the Cyt × Nuc × N interaction in our experiment: in N starvation conditions, their accumulation in cytolines was more enhanced than in parental lines. This suggests that in cytolines mitochondria participate in modulating the response to N starvation compared to what occurs in the natural parental lines. The enrichment in genes encoding nucleus-located proteins among DEGs in Cyt × Nuc interaction (Table 1) suggests that at least part of this modulation in the new cyto-nuclear combinations is achieved through the modulation of the transcriptional response.

#### 2.2.4. Disruption of Cyto-Nuclear Coadaptation Modulated the Transcriptional and Translational Regulation by N Starvation

The N/C metabolic balance is a crucial issue in N starvation response, as C skeletons produced by photosynthesis are no longer recruited for nitrogen assimilation. The reprogramming of metabolism in order to maintain N/C homeostasis under N starvation involves regulations at many different levels: transcriptional, translational, post-translational as protein degradation or activation/inhibition by phosphorylation or redox modification, metabolite compartmentalization... [11]. Despite many of these levels of regulation being out of reach of our experimental results, we were able to monitor gene expression regulatory response to N starvation, and how it is modulated in the disruption of cyto-nuclear coadaptation.

**Figure 9:**
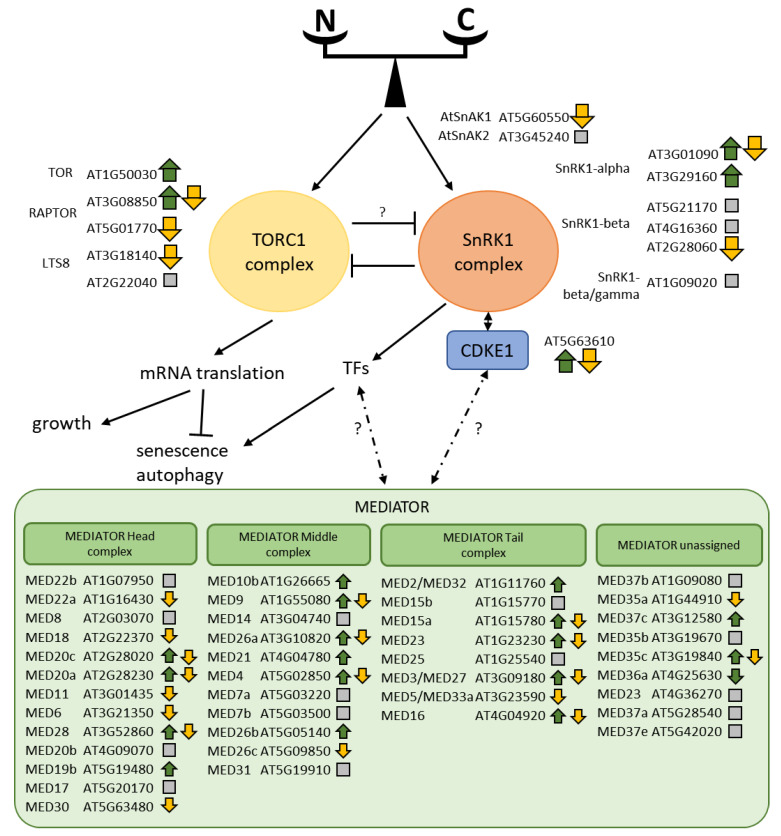
Effects of N starvation and disruption of cyto-nuclear coadaptation on the expression of genes coding the TOR SnRK1, and MEDIATOR complex subunits. ANOVA results are symbolized as in Figure 5. TFs, transcription factors.

It is widely admitted that the central TOR/SnRK1 kinases regulatory hub integrates N/C/energy signaling pathways in plant cells, as in other eukaryotes [11,88,89]. TOR and SnRK1 complexes are considered to act antagonistically. SnRK1 inactivates TOR through phosphorylation of its RAPTOR subunits, and several evidences suggested a reciprocal regulation of SnRK1 by TOR [88]. Recent study reported that abiotic stresses, such as salt, cold and osmotic stresses, regulate TOR complex transcript levels and activity [90]. In our experiment, expression of genes encoding TOR (*AT1G50030*), RAPTOR3G (*AT3G08850*) and the SnRK1 catalytic (*AT3G01090* and *AT3G29160*) subunits were induced by N starvation, while other (regulatory) subunits of the complexes were repressed in cytolines compared to parents (Figure 9). CDKE1, which interacts with SnRK1 [91], is a putative integrator of retrograde signals from organelles, and is also induced by N starvation and repressed by the disruption of cyto-nuclear coadaptation in our experiment. As both TOR and SnRK1 complexes are regulated post-translationally by phosphorylation, it is difficult to evaluate the impact of these transcriptional changes on their activities. Interestingly, it has been proposed that CDKE1 relays the SnRK1 nutrition signals to promoter-bound TFs and RNA polymerase II through interaction with the plant MEDIATOR complex [17,92]. We examined the responses of 41 subunits of the plant MEDIATOR [93] in our experiment and found that 18 were affected by N starvation (17 up-regulated, one down-regulated), and 19 were less expressed in cytolines compared to parents (Figure 9). Eleven MEDIATOR subunits were affected by both N starvation and Cyt × Nuc (Figure 9), and two by the Cyt × Nuc × N interaction (MED26c and MED15a). These results suggest that the new genomic context of cytolines is likely to modulate the response to N starvation through its transcriptional regulatory component in our experiment.

The GO analysis of DEGs also highlighted the importance of transcriptional regulation in response to N starvation. From the platform PlantRegMap [94], we extracted the whole list of transcription factors described in *Arabidopsis* and examined their responses in our experiment. Thus, 761 TFs were affected by N starvation, among which 309 were also differentially expressed in cytolines compared to parents, and 26 responded to the Cyt × Nuc × N interaction. Several well-known genes involved in nitrate signaling pathways, such as *ANR1* (*AT2G14210*), *LBD39* (*AT4G37540*), *TCP20* (*AT3G27010*), *TGA1* (*AT5G65210*), *TGA4* (*AT5G10030*), *NAC4 (AT5G07680*), *NIGT1.1* (*AT1G25550*) *NIGT1.2* (*AT1G68670*) and *NIGT1.3* (*AT3G25790*), were regulated by N starvation. *SPL9* (*AT2G42200*), *LBD39, NIGT1.2* and *TCP20* were under Cyt × Nuc interaction. Indeed, *TCP20* was also affected by the Cyt × Nuc × N interaction since *TCP20* was not induced by N starvation in the cytoline *52CV* unlike in the other lines. This suggests that the nitrate response mediated by *TCP20* was not activated in this cytoline. The regulation of several TFs is coherent with the strong transcriptional response to N starvation previously described [95], and indicates that the transcriptional signature of the new genomic combinations of cytolines affects a part of this complex regulatory system.

Translation was the most enriched biological process in the DEGs repressed by N starvation, and the second most enriched one in the DEGs for which the effect of N starvation on translation was reinforced in cytolines compared to parents (i.e., affected by both N starvation and Cyt × Nuc in the same direction). Ribosomal proteins (RP), which account for around 5% of total N nitrogen in leaves [33], are a source of mobilized nitrogen when plants are N starved. Plant cells are known to coordinate eukaryotic ribosomal protein (cyto-ribosome) and prokaryotic ribosomal protein (chloro-ribosome and mito-ribosome) productions [96]. The composition, structure and organization of the three cellular ribosomes have been studied in detail in *Arabidopsis* [97,98,99]. We listed 245 genes encoding cyto-ribosome RPs, 66 genes for chloro-ribosome and 93 genes for the mito-ribosome proteins (Table S10).

**Figure 10:**
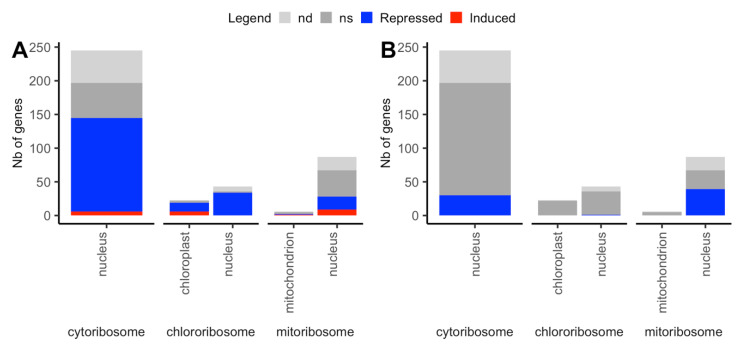
Transcriptional responses of genes encoding the three cellular ribosomes. (**A**) Response to N starvation. (**B**) Response to the disruption of cyto-nuclear coadaptation. Colors show significant genes up- and down-regulated by N starvation in red and blue; non-significant (ns) in dark grey and non-detected (nd) in light grey.

Figure 10 shows the transcriptional responses of RP genes to N starvation and to the disruption of cyto-nuclear coadaptation. We observed a selective transcriptional response to N starvation among the three categories of ribosomes. More than 70% of genes coding for cytoplasmic RPs, 59% of cp genes and 94% of nuclear genes coding for cp RPs were down regulated under N starvation, while around 40% of nuclear and mt genes coding for mt RPs were affected by the N stress. Oppositely, 58% of nuclear genes coding for mitochondrial RPs were less expressed in cytolines than in parents, whereas the other ribosomes were poorly affected by the disruption of cyto-nuclear coadaption. Only four DEGs coding for RPs were affected by the Cyt × Nuc × N interaction: *RPL28e* (*AT4G29410*), cp *RPL36* (*ATCG00760*), and cp *RPS8* (*ATCG00770*) were less repressed by N starvation in cytolines than in parents, while mt *RPS18* (*AT1G07210*) was less induced by N starvation in cytolines than in parents, which suggests that the disruption of cyto-nuclear coadaptation tended to attenuate the transcriptional response to N starvation. Interestingly, the gene encoding the PPR (Pentatrico Peptide Repeat) PGR3 (*Proton Gradient regulation 3*; *AT4G31850*), recently shown to stimulate RPS8 translation [100], was down regulated by N starvation in our experiment, further indicating the repression of protein translation in the chloroplast in this condition. At the protein level, 6 out of the 41 quantified cytosolic RPs and 34 out of the 36 quantified cp RPs were less accumulated in N starved plant, in accordance with the down-regulation of their respective mRNA (no mt RP was quantified in the experiment). This confirmed the strong effect of N starvation on protein synthesis in the cytosol and chloroplasts.

Remarkably, 27% of cp RP genes, 20% of mt RP genes and 13% of nuclear genes encoding mt RPs were enhanced by N starvation. The opposite variation of regulation of some RP genes by N nutrition suggested that the ribosomal composition changed in the organelles upon nutrition stress. It has been reported that RP genes were regulated by various environmental conditions, which caused changes in ribosome compositions [101,102]. Following the ribosomal filter hypothesis proposed by Mauro and Edelman [103], the ribosomes may not be simply translation machines but they could also act as regulatory elements that differentially translate particular mRNAs. The modification of ribosome composition in mitochondria and chloroplasts may favor the distinctive responses to N stress in the different cellular compartments.

Altogether, these results indicate that the translation machineries were down regulated in the cytoplasm and plastids but were maintained in mitochondria under N starvation, while the mito-ribosome was more sensitive to the new genomic situation of cytolines. They also suggest that some RPs could participate in regulation of the translated mRNAs filtering during the N starvation response.

## 3. Conclusions

This study was designed to evaluate whether the coadaptation between nuclear and organellar genomes of plant cells contributes to the molecular response of the plant to a nutrient stress, to what extent and at which level. We addressed the effect of the disruption of cyto-nuclear coadaptation on the plant response to N starvation by examining the gene expression, protein amount and metabolite accumulation responding to the Cyt × Nuc × N interaction effect or responding to both the N and the Cyt × Nuc interaction effects. We highlight here the main results of the study:

(i) Unexpectedly, a large excess of genes affected by the disruption of cyto-nuclear co-adaptation were repressed in the cytolines compared to the parental lines, which suggests that the transcriptional response in the cytolines to their non co-adapted genomic situation was a repression of gene expression.
(ii) The disruption of cyto-nuclear coadaptation down-regulated nuclear genes encoding mitochondrial proteins, but did not modify the expression of mitochondrial genes, indicating a selective transcriptional response of genes encoding mitochondrial proteins according to their genomic localization.
(iii) There is a clear modulation of the molecular response to N starvation in cytolines compared to their parents, mainly at the levels of gene expression and metabolite accumulation, and in particular for biological processes occurring within mitochondria and chloroplasts.
(iv) Autophagy, ubiquitin/26S proteasome pathway, recycling of purines, which are important physiological responses to N starvation, were attenuated in cytolines compared to their parents.
(v) The primary C and energy metabolism responses to the N stress were also modulated in cytolines, suggesting differences in the energy homeostasis and C skeleton management.
(vi) The new genomic situation of cytolines might be managed through the TOR/SnRK1 regulatory hub, regulatory transcriptional factors and down regulation of genes coding for mitochondria located proteins.
(vii) While chloroplasts are clear targets of the response to N starvation, mitochondria appeared also to be at the frontline to cope with the non-coadapted cytonuclear combinations of cytolines.

More dedicated studies to specific pathways are now needed to get a more comprehensive picture of these modulations.

## 4. Materials and Methods

### 4.1. Genetic Material

Seeds for all genotypes were obtained from the Versailles *Arabidopsis* Stock Centre [104]. The construction of cytolines, which carry the cytoplasmic genomes from one natural line and the nuclear genome from another one was described in [29].

### 4.2. Culture and production of Samples

Seeds were surface sterilized by using ethanol–‘bayrochlor’ (95/5%, *v*/*v*) prior to stratification in water at 4 °C for three  days. Each seed was sown using a toothpick on the top of one cut Eppendorf tube filled with 0.7% agar. Tubes were inserted into 96-wells trays filled with demineralized water. After 3 days in the darkness at 4 °C, the trays were transferred to a growth chamber in short days with 21 °C day and 17 °C night temperatures. Relative humidity in the growth chamber was 65%. The photon flux density was 165 μmol × m^−2^ × s^−1^. On the seventh day of growth, seedlings were transferred to plastic tanks. Each genotype was represented by eight plants per nutrition condition. These plastic tanks were filled with 33.0 L of nutrient solution. The plants were cultivated hydroponically for 42 days (the entire vegetative growth). A short photoperiod (8 h day) was chosen to prevent early flowering.

One set of plants was fed on complete nutrient solution containing 4 mM nitrate as the N source (2 mM KNO_3_, 1 mM Ca(NO_3_)_2_, 0.15 mM KH_2_PO_4_, 0.15 mM MgSO_4_, 0.3 mM CaCl_2_) for 42 days. In the second condition, a set of starved plants that were maintained on the complete nutrient solution for 35 days were subjected for 1 week to a complete N starvation with 0 mM N solution (0.15 mM KH_2_PO_4_, 0.5 mM K_2_SO_4_, 0.15 mM MgSO_4_, 0.18 mM CaCl_2_). All nutrient solutions contained microelements (15 μM MnSO_4_, 10 μM H_3_BO_3_, 3 μM ZnSO_4_, 0.01 μM CuSO_4_, 0.1 μM Na_2_MoO_4_, 22.3 μM Na-EDTA, 22.4 μM FeSO_4_, 0.01 μM CoCl_2_, and 0.5 μM KI). Solutions were renewed once during each week of culture up to harvest.

Three successive cultures were carried out to produce three independent replicates of the experiment. Harvested samples were immediately frozen. The rosettes from four plants of each genotype and each condition were pooled, thus producing two pools by experiment. Samples were ground with the help of steel bullets in a shaking grinder. Fresh matters, nitrate and free amino acid content measurements were performed on these pools (Figure 1 and Figure S1). In order to obtain enough material for performing the multi-omics measures, both pools from each biological repetition were grouped before the production of transcriptomic, proteomic and metabolomic data.

### 4.3. Nitrate and Free Amino Acids Contents

An aliquot of the obtained powder was weighed and used for extraction of metabolites. A two-step ethanol-water extraction was used, as described in Loudet et al. [105]. The first step consisted in a 25-min extraction at 80 °C using 500 μL of 80% (*v*/*v*) ethanol, whereas the second step completed the extraction by using 500 μL of double-distilled water at 80 °C for 20 min. Supernatants obtained from the two extractions were collected and put together in a well of a 2 mL 96 well plate and dried overnight in a speed-vacuum machine. Samples were then dissolved in 600 μL of double-distilled water and frozen at −20 °C before analysis. Pellets obtained after removing supernatants were dried for one night at 40 °C and used for starch extraction.

#### 4.3.1. Nitrate Content

Extracts were evaporated and diluted in water before analysing them for nitrate contents. The method used was as described by Miranda et al. [106]. The reactant was prepared by dissolving vanadium III chloride (0.5 g), N-(1-naphthyl)ethylenediamine (0.01 g), and sulphanilamide (0.2 g) in HCl (0.5 M); 1 mM NaNO_3_ was used as standard. After loading the plate with samples (100 μL), an equal volume of reactant was added to each well and the reaction was carried out at room temperature for 5–6 h. The absorbance at 540 nm was measured using a spectrophotometer (Labsystem iEMS Reader MF, MTX Lab Systems, Bradenton, Florida, USA) and used to estimate the nitrate content in nmol mg^−1^ FM.

#### 4.3.2. Free Amino Acids Content

The same extracts were subjected to an evaluation of free amino acid content using glutamine as a standard Rosen [107]. Briefly, ninhydrin color reagent was first made by dissolving 0.3 g of ninhydrin (Sigma-Aldrich Chimie, Lyon, France) in 10 mL of methyl cellosolve (Sigma-Aldrich Chimie, Lyon, France). Cyanide acetate reagent was then prepared by mixing Na-acetate buffer (25 mL, 2.5 M, pH 5.2) with the KCN (10 mM) solution 2/100 (*v*/*v*) just prior to reaction. In a 96-well (2 mL) plate containing 200 μL of sample (diluted), 100 μL of ninhydrin color reagent followed by 100 μL of cyanide acetate reagent were added. The plate was shaken and heated for 15 min at 100 °C. After cooling, 1 mL of isopropanol (50% *v*/*v*) was added to the wells of the plate and mixed well. Absorbance was read at 570 nm on a spectrophotometer. This result was used to calculate the amino acid content in nmol mg^−1^ FM using a glutamine 4 mM dilution set as calibration standard.

### 4.4. Transcriptomic Data

Total RNA extracted from the ground samples following TRI-reagent protocols (Sigma-Aldrich, St Louis, MO, USA) and treated twice with RNase-free DNase I (Thermo Fisher Scientific, Les Ulis, France) was finally purified using NucleoSpin RNA kits (Macherey-Nagel, Hoerdt, France). RNA samples were checked for quality on a bioanalyser (Agilent, Les Ulis, France), quantified with a Quant-iT RiboGreen RNA Assay Kit (Thermo Fisher Scientific, Les Ulis, France).

Microarray analyses were carried out on the INRA transcriptomic platform at the IPS2 (Orsay, France) using CATMAv6.1 (Roche-NimbleGen technology, Madison, WI, USA) arrays. High density CATMAv6.1 microarray slides contained, per chamber, 270,000 primers representing all *Arabidopsis* genes, i.e., 30,834 probes referring to the TAIRv8 annotation (including 476 probes of mitochondrial and chloroplast genes) (www.arabidopsis.org/), plus 1289 probes corresponding to EUGENE software predictions, 5352 probes corresponding to repeated elements, 658 probes for miRNAs, 342 probes for other non-coding RNAs (rRNAs, tRNAs, snRNAs, soRNAs) and finally 36 control probes. Each long primer is triplicate in each chamber for robust analysis. For each comparison, one technical replicate with fluorochrome reversal was performed for each biological replicate (i.e., four hybridizations per comparison). cRNAs were labeled with Cy3-dUTP or Cy5-dUTP (Perkin-Elmer-NEN Life ScienceProducts, Courtaboeuf, France). The samples were normalized as described in [108] and then a normalized intensity per gene for each sample was calculated using the method described in [109].

Microarray data from this study were deposited into the CATdb database [110,111] (tools.ips2.u-psud.fr/CATdb/, Project: BioAdapt2011_Cytopheno) and into the Gene Expression Omnibus (GEO) repository [112] at the National Center for Biotechnology Information (NCBI) (accession number GSE144597) according to the “Minimum Information About a Microarray Experiment” standards [113].

The RT-qPCR analysis of organellar transcripts was carried out as described in [114,115], except that the qPCR was performed on a CFX384 real-time System (BioRad, Marnes-la-Coquette, France) with the SybrGreen Premix Ex Taq kit (Tli RNaseH plus, Takara, Japan).

### 4.5. Proteomic Data

For each sample, 200 mg of fresh rosette leaves were ground to a fine powder with liquid nitrogen in a mortar. Total proteins were then extracted with a TCA-acetone protocol according to Méchin et al. [116]. The dry pellets of precipitated proteins were solubilized in a specific buffer containing 6 M urea, 2 M thiourea, 10 mM DTT, 30 mM Tris-Hcl pH 8.8 with 0.02% (*w*/*v*) ProteaseMAX (Promega, Madison, WI, USA), at room temperature for 1.5 h. We treated 3 µL of sample, corresponding to 60 µg of total proteins, with 1µL of 0.5M DTT (20 min at 56 °C) and 2 µL of 0.55 M iodoacetamide for reduction and alkylation process before launching the digestion step with 10 µL of trypsin at 0.2 µg/µL for 3 h at 37 °C. We added 1 µL of 1% ProteaseMAX in the reaction medium. As a last step of preparation, samples were desalted by solid phase extraction with 3MTM EmporeTM C8 purification discs (Sigma-Aldrich, St Louis, MO, USA).

LC-MS/MS analyses were run in random order on a Nano-LC U3000 RSLC system (Thermo-Fisher) coupled to a LTQ Orbitrap Velos mass spectrometer (Thermo-Fisher). We injected 6 µL of samples to be separated on a 50-cm C18 column (Acclaim Pepmap C18, 75 µm internal diameter, Thermo-Fisher). A linear gradient of 112 min increasing from 4% to 70% of B (90% acetonitrile, 0.08% formic acid) was applied with a flow rate of 300 nL/min. The MS scan resolution was set at 60,000 between 400 and 2000 m/z. The MS/MS fragmentation was performed by CID on the 8 top precursors of the MS1 spectrum.

MS raw data were converted into mzXML files thanks to MS Convert (ProteoWizard) to be analyzed with the tools developed and provided by the PAPPSO platform (INRAE, Gif-sur-Yvette, France). The protein identifications were done thanks to the X!Tandem software by first querying the MS/MS data against the TAIR10_pep_20101214 fasta database and a contaminant one for trypsin and keratins. Proteins were then filtered on a basis of at least two corresponding peptides with an E-value smaller than 0.001 and a protein log(E-value) smaller than −4. Relative peptide quantification by peak-area integration on XICs (eXtracted Ion Chromatogram) was performed using MassChroQ software. Relative protein abundance was then obtained thanks to an R script using the package MassChroqR for filtering, normalization, imputation of missing data and calculation of proteins abundance from peptides data. This calculation was done by summing the intensities of at least two specific (non-redundant) and reproducible peptides by quantified protein. Finally, this pipeline for quantitative proteomic provided us with a table of 665 proteins quantified on the 24 samples of the project, ready for statistical analysis.

### 4.6. Metabolomic Data

Extraction and gas chromatography–mass spectrometry (GC-MS) analyses were performed as described by Tcherkez et al. [117]. The relative levels of metabolites were determined in an untargeted manner. Leaf samples (100 mg of fresh powder) were ground in liquid nitrogen using a Mixer Mill MM400 (Retsch, Germany) for 2 min at a frequency of 30 Hz and then in 2 mL of 80% methanol in which ribitol (100 µmol L^−1^) was added as an internal standard. After centrifugation, aliquots of each extract (0.2 mL) were spin-dried under vacuum. The extracts were dissolved with methoxyamine (in pyridine) and N-methyl-N (trimethyl-silyl) trifluoroacetamide (MSTFA). The derivatization mixture was then incubated for 2 h at room temperature. Before loading into the GC autosampler, a mix of a series of eight alkanes (chain lengths: C10–C36) was included. Analyses were performed by injecting 1 µL in splitless mode at 230 °C (injector temperature). Gas chromatography coupled to time-of-flight mass spectrometry was performed on a LECO Pegasus III with an Agilent (Massy, France) 6890N GC system and an Agilent 7683 automatic liquid sampler. The column was an RTX-5 w/integra-Guard (30 m × 0.25 mm internal diameter + 10 m integrated guard column; Restek, Evry, France). The chromatographic separation was performed in helium as a gas-carrier at 1 mL min^−1^ in the constant flow mode and using a temperature ramp ranging from 80 to 330 °C between 2 and 18 min, followed by 6 min at 330 °C. Electron ionization at 70 eV was used and the MS acquisition rate was 20 spectra s^−1^ over the m/z range 80–500 as described by Weckwerth et al. [118]. Peak identity was established by comparison of the fragmentation pattern with MS available databases [119], using a match cut-off criterion of 700/1000 and by retention time using the alkane series as retention standards. The integration of peaks was performed using the Chroma TOF software, version 2.22 (Leco, Garges-lès-Gonesse, France). Because automated peak integration was occasionally erroneous, integration was verified manually for each compound in all analyses. As a quality control filter, samples were checked for the presence of a strong ribitol peak with a peak area of at least 35,000 and a deviation from the median internal standard peak area (for that GC/MS batch sequence) of less than 15% of the median value. Metabolite contents are expressed in arbitrary units (semi-quantitative determination). Peak areas determined using the LECO Pegasus software have been normalized to fresh weight and ribitol area (internal standard).

### 4.7. Statistical Analyses

Each type of accumulation was modeled with a three-way ANOVA as follows:

Y_ijkr_ = μ + C_i_ + N_j_ + A_k_ + CN_ij_ + CA_ik_ + NA_jk_ + CNA_ijk_ + E_ijkr_ (1)

where Y_ijkr_ is the normalized accumulation of a given entity (transcript, protein or metabolite) for a plant with cytoplasm i and nuclear j at nitrate supply k in replicate r, μ the global mean, C_i_ the cytoplasmic effect, N_j_ the nuclear effect, A_k_ the nitrate effect, CN_ij_ the cytoplasm × nucleus interaction effect, CA_ik_ the cytoplasm × nutrition interaction effect, NA_jk_ the nucleus × nutrition interaction effect, CNA_ijk_ the cytoplasm × nucleus × nutrition interaction effect, and E_ijkr_ are normally distributed zero-mean random errors. Parameters were estimated using the 24 available observations per biological entity.

Seven contrasts were considered to classify the quantified molecules as responsive to (i) the nitrogen supply across both cytoplasms and both nuclei, (ii) the nucleus origin across both nitrogen supplies and both cytoplasms, (iii) the cytoplasm origin across both nitrogen supplies and both nuclei, (iv) a cytoplasm × nucleus interaction effect across both nitrogen supplies (v) a cytoplasm × nitrogen interaction effect across both nuclei (vi) a nuclear × nitrogen interaction effect across both cytoplasms and (vii) a cytoplasm × nuclear × nitrogen interaction effect. The seven contrasts were calculated as follows:

(i) The nitrogen starvation effect = mean(all genotypes in nitrogen starvation) − mean(all genotypes in control nitrogen supply).
(ii) The nucleus effect = mean(genotypes with *Jea* nucleus in both nitrogen conditions) − mean(genotypes with *Ct-1* nucleus in both nitrogen conditions).
(iii) The cytoplasm effect = mean(genotypes with *Jea* cytoplasm in both nitrogen conditions) − mean(genotypes with *Ct-1* cytoplasm in both nitrogen conditions).
(iv) The cytoplasm × nucleus interaction effect = mean(both cytolines in both nitrogen conditions) − mean(both parental lines in both nitrogen conditions).
(v) The nucleus × nitrogen interaction effect = [mean(genotypes with *Jea* nucleus in nitrogen starvation) − mean(genotypes with *Jea* nucleus in control nitrogen supply)] − [mean(genotypes with *Ct-1* nucleus in nitrogen starvation) − mean(genotypes with *Ct-1* nucleus in control nitrogen supply)].
(vi) The cytoplasm × nitrogen interaction effect = [mean(genotypes with *Jea* cytoplasm in nitrogen starvation) − mean(genotypes with *Jea* cytoplasm in control nitrogen supply)] − [mean(genotypes with *Ct-1* cytoplasm in nitrogen starvation) − mean(genotypes with *Ct-1* cytoplasm in control nitrogen supply)].
(vii) The cytoplasm × nucleus × nitrogen interaction effect = [mean(both parental lines in nitrogen starvation) − mean(both parental lines in control nitrogen supply)] − [mean(both cytolines in nitrogen starvation) − mean(both cytolines in control nitrogen supply)].

*p*-values of the seven contrasts were adjusted altogether by the Benjamini-Hochberg method to control FDR. For a given contrast, an accumulation was declared different if its adjusted *p*-value was lower than 0.05. Although the p-value adjustment was calculated on the seven contrasts, we focused the interpretation on the effects (i), (iv) and (vii) (see Results and Discussion).

The scripts and datasets used for this analyses are accessible at [34], together with the results of the analyses.

### 4.8. Bioinformatics Analyses

#### 4.8.1. Exclusion of CATMA Probes Covering Polymorphisms Between Parental Accessions

The lists of polymorphisms in *Jea* and *Ct-1* compared to *Col-0* (TAIR10 Reference genome) were downloaded from the 1001 Genomes website [120] and converted into .vcf files. Unique ID were attributed for each polymorphism and the two lists were compared to extract polymorphisms between *Jea* and *Ct-1*. The position of CATMA probes were used to identify *Jea/Ct-1* polymorphisms on probe sequences, which could induce biases in hybridization efficiency. CATMA probes whose sequence contained a polymorphism between *Jea* and *Ct-1* were ruled out, which excluded 5629 genes from the DEG analysis.

#### 4.8.2. Enrichments in Subcellular Location of DEG Products

We used the SUBAcon locations of gene products (http://suba.live/, downloaded in January 2018 [121]. For each list of DEGs and each subcellular location, enrichment compared to the complete set of analyzed genes was tested using the phyper function in R. In some specified cases, the reference set was modified as indicated in Table 1 footnote.

#### 4.8.3. Enrichments in Biological Processes of DEGs

The assignment of protein functions was based on the TAIR GO categories from the aspect “biological process”. Enrichment analysis of GO categories was done in R (version 3.6, http://www.r-project.org) using the “elim” method from the topGO package [122] that is part of the Bioconductor project (version 3.10, http://www.bioconductor.org/). TopGO is a software package, which provides tools for testing GO terms by identifying and removing local dependencies between GO terms [123]. The elim algorithm iteratively removes the genes mapped to significant terms from higher-level GO terms. Fisher’s exact test was used for assessing the GO term significance. Over-representation of biological processes was calculated for 1) the DEGs up and down regulated under N starvation as compared to all transcripts identified in our dataset and predicted in TAIR10; 2) the DEG responsive to both N starvation and Cyt × Nuc intersection with common or opposite senses as compared to all up and down DEG by N starvation.

## Figures and Tables

**Table 1:** Predicted subcellular locations of differentially expressed gene (DEG)-encoded proteins.

Subcellular Location ^1^	DEGs in N Nutrition ^2^	DEGs in Cytolines vs. Parents ^2^	DEGs in Third Order Interaction Cyt × Nuc × N ^2^	DEGs in Both N Nutrition and Cyt × Nuc ^3^
Cytosol	2237 *	1234	96	769
Endoplasmic Reticulum	303	176	21	103
Extracellular	989	449	34	249
Golgi	281	218 *	32 *	**137 ^#^**
Mitochondrion	853	622 *	55	**370 ^#^**
Nucleus	3010 *	2230 *	154	**1405 ^#^**
Peroxisome	138	78	12	52
Plasma Membrane	1483 *	920 *	89	**610 ^#^**
Plastid	1294 *	598	75 *	370
Vacuole	238 *	105	8	68
Total	12255	7153	604	4437

^1^ according to SUBA4con. ^2^ * indicate enrichments compared to the dataset of analysed genes (*p* < 0.001). ^3^ # indicate enrichments compared to the dataset of DEGs in N starvation (*p* < 0.001).

## Supplementary Materials

The following are available online at https://www.mdpi.com/2223-7747/9/5/573/s1, Figure S1: Fresh matter and senescence of harvested plants, Table S1: List of AGI corresponding to differentially expressed genes under N starvation, Cyt × Nuc interaction and Cyt × Nuc × N interaction. Table S2: List of AGI corresponding to differentially accumulated proteins under N starvation, Cyt × Nuc interaction and Cyt × Nuc × N interaction. Table S3: List of differentially accumulated metabolites under N starvation, Cyt × Nuc interaction and Cyt × Nuc × N interaction. Table S4: Gene expression response of organelle stress for N starvation and cytonuclear coadaptation and the third order interaction effect Cyt × Nuc × N. Table S5: Gene expression response to N starvation, cytonuclear coadaptation and the Cyt × Nuc × N interaction of genes identified by Miclaus et al. 2016 as signatures of the retrograde signaling pathways in maize. Table S6: gene expression response of proteolysis complexes for N starvation, cytonuclear coadaptation and the third order interaction effect Cyt × Nuc × N. Table S7: gene expression response of photosynthetic complex subunits for N starvation, cytonuclear coadaptation and the third order interaction effect Cyt × Nuc × N. Table S8: gene expression response of genes involved in lipid metabolism for N starvation, cytonuclear coadaptation and the third order interaction effect Cyt × Nuc × N. Table S9: gene expression response of mitochondrial oxidative phosphorylation complex subunits for N starvation, cytonuclear coadaptation and the third order interaction effect Cyt × Nuc × N. Table S10: gene expression response of proteins constituting the three cellular ribosomes for N starvation, cytonuclear coadaptation and the third order interaction effect Cyt × Nuc × N.
