# Supplementary material for: The Consequences of a Disruption in Cyto-Nuclear Coadaptation on the Molecular Response to a Nitrate Starvation in Arabidopsis
Source: Plants (Basel). 2020 May 1;9(5):573. doi: 10.3390/plants9050573 (PMC7285260; doi:10.3390/plants9050573)
Supplement: Supplementary file 1 [file plants-09-00573-s001.zip › Suppfilesv2/Figure_S1-revV3.docx]

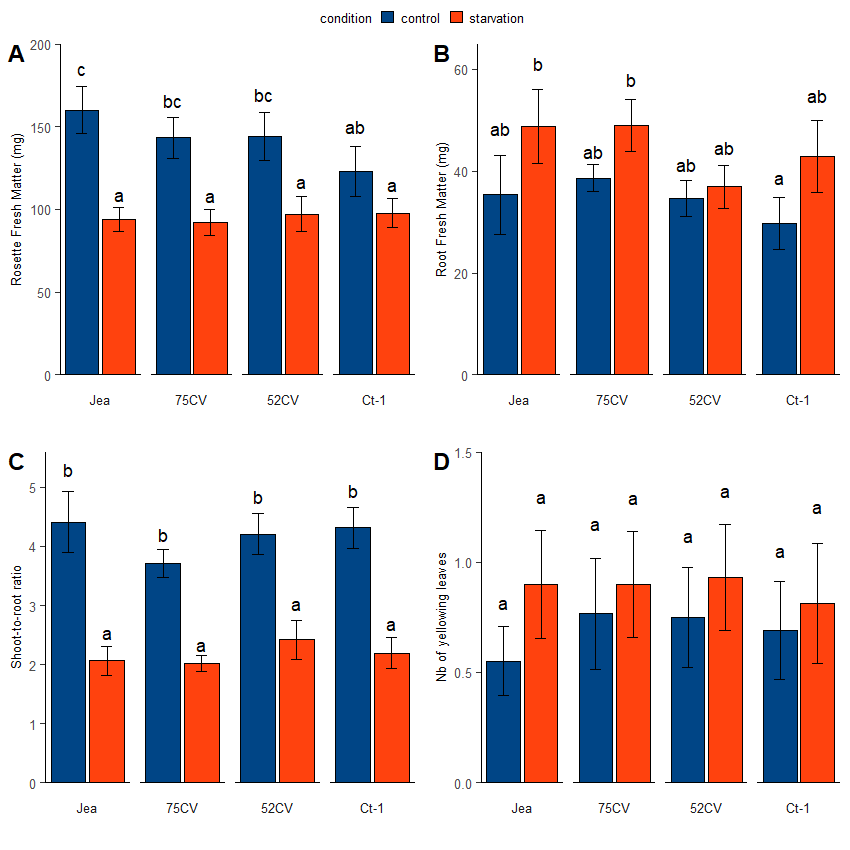


Figure S1: Fresh matter and senescence of harvested plants. Blue and red bars show genotype values for rosette FM (A), root FM (B), Shoot-to-root ratio (C) and leaf senescence (D), in the control and N starvation conditions respectively. Error bars are SE (n = 6: 2 pools of 4 plants x 3 experiments). Different letters indicate values significantly different (Tukey's test, p < 0.05).
